# Supplementary material for: Genome-Wide Association Studies of Seven Root Traits in Soybean (Glycine max L.) Landraces
Source: Int J Mol Sci. 2023 Jan 3;24(1):873. doi: 10.3390/ijms24010873 (PMC9821504; doi:10.3390/ijms24010873)
Supplement: Supplementary file 1 [file ijms-24-00873-s001.zip › ijms-2063410-supplementary.pdf]

Table S1. Descriptive statistics for seven root traits in 357soybean landraces.

Traits	Range	Mean	SD[a]	CV (%)[b]	Skewness	Kurtosis
DIAM.	0.404 - 0.666	0.537	0.046	8.686	0.111	-0.391
LAD	0.429 - 0.725	0.563	0.052	9.256	0.134	-0.133
LAL	0.109 - 0.379	0.216	0.032	15.120	1.104	2.996
LENGTH	703.330 - 3212.642	1766.263	422.533	23.922	0.281	-0.075
NF	1180.000 - 11941.670	5250.514	1692.208	32.229	0.380	-0.002
NT	622.670 - 3255.000	1777.584	525.085	29.539	0.368	-0.431
SA	124.148 - 491.667	294.381	66.226	22.497	0.184	-0.127

[a] Standard deviation; [b]Coefficient of variation

Table S2. Eigenvalue and proportion of principal components.

Principal Component	Eigenvalue	Variability (%)	Cumulative (%)
1	74.38	34.00	34.20
2	30.64	14.00	48.30
3	21.55	10.00	58.20
4	17.71	8.00	66.40
5	15.67	7.00	73.60
6	15.02	7.00	80.50
7	11.52	5.00	85.80
8	10.73	5.00	90.70
9	10.28	5.00	95.50
10	9.72	4.00	100.00

Table S3. List of putative candidate genes associated with significant SNPs from MLM model at the vicinity of (100 Kb either side).

**Trait**	**SNP**	**Chr**	***Gene Name***	**Start**	**End**	**Strand**	**Description**
**DIAM**	AX-90460045	Gm02	*Glyma.02g248800*	43611197	43611776	+	_
			Glyma.02g249000	43662205	43664226	-	Aspartyl proteases
			Glyma.02g249100	43676217	43678117	-	E3ubiquitin-proteinligasecip8
			Glyma.02g249200	43678739	43679865	-	_
			Glyma.02g249300	43682559	43685068	-	Glycerol-3-phosphateacyltransferase5-related
			Glyma.02g249400	43689781	43693616	-	Proteindnj-23-related
			Glyma.02g249500	43704884	43707125	-	Thaumatin family(thaumatin)
			Glyma.02g249600	43713516	43717718	-	Ribulosebis phosphate carboxylase/oxygenase activase, chloroplastic
			Glyma.02g249700	43723333	43724257	+	Lob domain-containing protein33
			Glyma.02g249800	43733046	43736212	-	At-hook motif nuclear localized protein 15
			Glyma.02g249900	43743444	43744190	+	_
			Glyma.02g250000	43747970	43755063	+	Guanyl-nucleotide exchange factor
			Glyma.02g250100	43762133	43762993	+	Transcriptional regulator superman
			Glyma.02g250200	43779470	43781548	+	Protein phosphatase2c3-related
			Glyma.02g250300	43783184	43785004	-	_
			Glyma.02g250400	43786923	43791698	-	Methyltransferase pmt10-related
			Glyma.02g250500	43797931	43798131	-	_
			Glyma.02g250600	43803483	43807640	+	PPR repeat(ppr)/ ppr repeat family (ppr_2)
	AX-90505093	Gm03	*Glyma.03g249500*	44562673	44568783	-	Proteingyg-1, isoform
	AX-90311611		*Glyma.03g248600*	44489655	44498277	-	Aldo/keto reductase

	AX-90308307<br>AX-90385325<br>AX-90442177<br>AX-90503377<br>AX-90345460		*Glyma.03g248700*	44499588	44502884	-	Aldo/keto reductase
			Glyma.03g248800	44506460	44512186	+	Zein-binding(zein-binding)
			Glyma.03g248900	44513489	44514230	+	_
			Glyma.03g249000	44521820	44526089	+	Grf1-interactingfactor1
			Glyma.03g249100	44529232	44529956	-	Vq motif (vq)
			Glyma.03g249200	44540832	44551676	+	Myosin-11-related
			Glyma.03g249300	44553517	44555047	-	F-box associated ubiquitination effector family protein
			Glyma.03g249400	44557289	44557836	-	_
			Glyma.03g249600	44569709	44572013	-	Profilin(profilin)
			Glyma.03g249700	44581032	44582153	-	C2domain(c2)
			Glyma.03g249800	44588637	44595274	+	Setdomain protein
			Glyma.03g249900	44595527	44597546	-	Caleosin-related family protein-related
			Glyma.03g250000	44599051	44603637	+	Myb family transcription factor-related
			Glyma.03g250100	44604344	44608040	+	Peroxisome assembly protein12
			Glyma.03g250200	44607834	44610091	-	_
			Glyma.03g250300	44611777	44616698	+	Alpha-1,6-fucosyltransferase
			Glyma.03g250400	44616824	44621636	-	Ankyrin repeat-containing protein
			Glyma.03g250500	44625641	44630894	+	Swib/mdm2,plus-3andgyfdomain-containingprotein-related
			Glyma.03g250600	44634084	44635415	+	Myb-like DNA-binding protein
			Glyma.03g250700	44637659	44643389	+	Chloroplastj-likedomain1-containingprotein
			Glyma.03g250900	44660923	44662144	+	Protein of unknown function (duf640)
			Glyma.03g251000	44667735	44668730	+	_
	AX-90385554	Gm03	*Glyma.03g253500*	44903910	44910278	-	Violaxanthin de-epoxidase, chloroplastic
			Glyma.03g253600	44909846	44913008	+	Non-specific serine/ threonine protein kinase/threonine-specific protein kinase
			Glyma.03g253700	44915061	44916435	+	Domain of unknown function (DUF4228)

	AX-90513850<br>AX-90502675<br>AX-90329829<br>AX-90504776<br>AX-90434642<br>AX-90416589<br>AX-90426968	Gm06	*Glyma.06g126000*	10293511	10313877	+	RNA recognition motif (rrm_1)/ peptidasefamilym41(peptidase_m41)/ relatedsubfamily)(aaa_5)//rnarecognitionmotif.(a.k.a.rrm,rbd,orrnpdomain)(rrm_5)
			Glyma.06g126100	10315437	10317265	-	Abscisic acid receptor PYl10-related
			Glyma.06g126200	10329274	10330451	+	_
			Glyma.06g126300	10331312	10341252	-	Trehalose-6-phosphate synthase
			Glyma.06g126400	10356650	10362151	-	_
			Glyma.06g126500	10367536	10373426	-	Proteindhs-1
			Glyma.06g126600	10376446	10384391	+	Cyclicnucleotide-gatedionchannel1
			Glyma.06g126700	10384771	10389578	-	Spermine synthase/spermidine amino propyl transferase
			Glyma.06g126800	10400498	10401860	-	F-box domain(f-box)
			Glyma.06g126900	10403126	10408307	-	3-oxoacyl-[acyl-carrier-protein] synthase-like protein
			Glyma.06g127000	10409463	10415886	+	_
			Glyma.06g127100	10419701	10425340	+	Protein of unknown function (duf668)
			Glyma.06g127200	10423873	10430156	-	Phosphomevalonatekinase
			Glyma.06g127300	10432974	10434886	-	Ringfingerdomain (zf-ring_2)/wall-associatedreceptorkinasegalacturonan-binding(gub_wak_bind)//wall-associatedreceptorkinasec-terminal(wak_assoc)
			Glyma.06g127400	10435277	10448595	-	_
			Glyma.06g127500	10462640	10469529	+	Splicing factor3 a subunit 3
			Glyma.06g127600	10474884	10482574	-	_
			Glyma.06g127700	10486250	10492229	+	Plastid-lipid-associated protein14, chloroplastic-related
	AX-90524509<br>AX-90332250	Gm14	*Glyma.14g197300*	46239260	46240644	+	Proteinlight-dependentshorthypocotyls5
			Glyma.14g197400	46269392	46271494	-	Apoprotein3, mitochondrial
			Glyma.14g197500	46280214	46282933	+	Ferroxidase/hephaestin

			Glyma.14g197600	46288254	46290941	+	Cinnamoyl-coareductase-likeprotein
			Glyma.14g197700	46291986	46298337	+	Transcriptioninitiationfactortfiidsubunit15b
			Glyma.14g197800	46303686	46308920	+	_
			Glyma.14g197900	46327072	46328700	+	Cct motif (cct)
			Glyma.14g198000	46334117	46336863	+	Udp-glycosyl transferase 87a2
			Glyma.14g198100	46337677	46340654	+	Glucosyl/glucuronosyl transferases
			Glyma.14g198200	46342657	46344315	-	Exocyst complex protein exo70
			Glyma.14g198300	46345968	46346877	+	Glucosyl/glucuronosyl transferases
			Glyma.14g198400	46347605	46349378	-	Exocyst complex protein exo70
			Glyma.14g198500	46352163	46354775	-	Exocystcomplexproteinexo70
			Glyma.14g198600	46360801	46362778	+	Udp-glycosyltransferase87a1
			Glyma.14g198700	46367820	46374604	+	Serine/threonine protein phosphatase
			Glyma.14g198800	46375963	46379746	-	Mitochondrial carrier protein mrs3/4
			Glyma.14g198900	46404283	46407292	-	Laccase-13-related
			Glyma.14g199000	46413031	46419952	+	Sortingnexin
			Glyma.14g199100	46421954	46422457	+	Exocystcomplexproteinexo70
			Glyma.14g199200	46425482	46428141	+	_
			Glyma.14g199300	46433103	46433444	+	_
			Glyma.14g199400	46435759	46438392	-	Diseaseresistanceproteinrpp13-related
	AX-90367887<br>AX-90402282<br>AX-90468047	Gm18	*Glyma.18g300200*	57789188	57820853	+	Callosesynthase3
			Glyma.18g300300	57823394	57831151	+	Wdsam1protein
			Glyma.18g300400	57831381	57834152	-	Oligo peptide transporter-related
			Glyma.18g300500	57841316	57843280	+	Pirin
			Glyma.18g300600	57844897	57846271	-	Gtpaserab11/ypt3, small g protein super family
			Glyma.18g300700	57852461	57859687	+	Signalpeptidepeptidase-like3-related
			Glyma.18g300800	57860162	57861657	+	_
			Glyma.18g300900	57867768	57869200	-	Auxin responsive protein (auxin_inducible)
			Glyma.18g301000	57890959	57891781	+	Non-specific serine/threonine protein kinase

			Glyma.18g301100	57894037	57895910	-	Bi directional sugar transporter sweet15
			Glyma.18g301200	57903571	57906434	-	Bi directional sugar transporter sweet15
			Glyma.18g301300	57913464	57915469	-	_
			Glyma.18g301400	57915964	57916724	-	_
			Glyma.18g301500	57926526	57928866	+	No apical meristem (nam)protein
			Glyma.18g301600	57939468	57945328	+	BAR domain
			Glyma.18g301700	57945537	57947130	-	Brassino steroid insensitive1-associated receptor kinase1-related
			Glyma.18g301800	57950394	57950985	+	_
			Glyma.18g301900	57953323	57956369	-	Lipopolysaccharide-induced transcription factor regulating tumor necrosis factoralpha
			Glyma.18g302000	57965322	57969597	+	Bomb/kira proteins
			Glyma.18g302100	57988925	57992117	+	Cdp-diacylglycerol--glycerol-3-phosphate3-
			Glyma.18g302200	57998307	58011231	+	Histone-lysinen-methyl transferase ASHR3
			Glyma.18g302300	58014611	58017908	+	65-k damicrotubule-associated protein 6-related
**LAD**	AX-90345460	Gm03	*Glyma.03g251100*	44673965	44702804	-	Exportin-4
			Glyma.03g251200	44703737	44706084	-	PPR repeat family (ppr_2)
			Glyma.03g251300	44708643	44717310	+	Phosphatase 2a regulatory subunit-related
			Glyma.03g251400	44718734	44719931	+	Non-specific serine/ threonine protein kinase/ phosphoenol pyruvate carboxy kinase (atp)
			Glyma.03g251500	44723994	44729654	-	Serine/threonine-protein phosphatase PP1 isozyme 2-related
			Glyma.03g251600	44735415	44736486	-	Peptidyl-prolylcis-trans isomerase cyp18-3-related
			Glyma.03g251700	44736493	44741875	-	Ethylene response sensor 2-related

			Glyma.03g251800	44744746	44751071	-	At hook motif DNA-binding family protein-related
			Glyma.03g251900	44752444	44755159	-	Associate of c-mycamy-1
			Glyma.03g252000	44756482	44767598	-	Suppressor of actin mutations 2/ vacuolar protein sorting 52
			Glyma.03g252100	44768483	44772056	+	Single-stranded DNA-binding protein why2, mitochondrial
			Glyma.03g252200	44773982	44778842	-	Duf 239, (duf4409)
			Glyma.03g252300	44781815	44809556	-	Translational activator gcn1
			Glyma.03g252400	44813173	44813557	+	_
			Glyma.03g252500	44819920	44825163	+	Gdslesterase/lipaseltl1
			Glyma.03g252600	44830497	44833487	+	Zincfingerfyvedomaincontainingprotei n
			Glyma.03g252700	44841960	44844706	+	Gdslesterase/lipaseltl1
			Glyma.03g252800	44849419	44851672	+	Zinc finger fyve domain containin gprotein
			Glyma.03g252900	44853430	44853922	+	Histidine kinase/proteinkinase(histidine)
			Glyma.03g253000	44856721	44878696	+	P53 inducible protein pir121
			Glyma.03g253100	44880486	44882438	+	Beta catenin-related armadillo repeat-containing
			Glyma.03g253200	44887335	44889341	-	Late embryogenesis abundant3 (lea3) family protein
			Glyma.03g253300	44888518	44892973	+	U1small nuclear ribonucleo protein
			Glyma.03g253400	44899608	44901536	-	_
	AX-90426968	Gm06	*Glyma.06g127800*	10493847	10506001	-	Histone-lysinen-methyl transferase atx4-related
			Glyma.06g127900	10510306	10515402	-	Cct motif family protein
			Glyma.06g128000	10534942	10537640	-	Nuclear migration protein
			Glyma.06g128100	10538914	10540591	+	Oxido reductase, 2og-feii oxygenase family protein
			Glyma.06g128200	10543911	10545747	+	Flavonolsynthase
			Glyma.06g128300	10546528	10547630	+	_

			Glyma.06g128400	10546758	10548460	+	Alpha/beta-hydrolases superfamily protein
			Glyma.06g128500	10564808	10569476	+	Pectin esterase/ pectin esterase inhibitor 34-related
			Glyma.06g128600	10572823	10578723	-	Dipeptidyl-peptidaseiv/xaa-pro-dipeptidyl amino peptidase
			Glyma.06g128700	10581580	10588773	-	Calcineurinb-likeprotein4
			Glyma.06g128800	10598708	10598973	-	_
			Glyma.06g128900	10600212	10602821	+	Pprrepeat (ppr)/ pprrepeat (ppr_1)/ ppr repeat family (ppr_2)
	AX-90481233	Gm12	*Glyma.12g192800*	35434955	35439253	-	Multi-copperoxidase
			Glyma.12g192900	35440475	35444708	-	_
			Glyma.12g193000	35450459	35456613	-	Arogenatedehydratase/carboxy cyclo hexadienyl dehydratase
			Glyma.12g193100	35463673	35472010	+	_
			Glyma.12g193200	35473199	35474392	+	_
			Glyma.12g193300	35478288	35482335	+	Myb-like DNA-binding protein
			Glyma.12g193400	35488883	35491610	-	_
			Glyma.12g193500	35498865	35503832	-	Alcohol dehydrogenase related
			Glyma.12g193600	35512904	35517589	-	Beta-1,3-galactosyl transferase 2-related
			Glyma.12g193700	35543782	35544619	-	_
			Glyma.12g193800	35551596	35559129	+	Aconitatehydratase/citrate (isocitrate) hydro-lyase
			Glyma.12g193900	35561363	35561746	-	_
			Glyma.12g194000	35567230	35571578	+	Multidrug resistance protein
			Glyma.12g194100	35577962	35583881	+	Glutamate receptor3.6-related
			Glyma.12g194200	35592309	35598542	+	Glutamate receptor3.4-related
			Glyma.12g194300	35602347	35603796	-	_
			Glyma.12g194400	35606060	35613310	-	Homeo box domain (homeobox)/start domain
		Gm13	*Glyma.13g162500*	27785225	27788045	-	_
			Glyma.13g162000	27766440	27766796	+	_

			Glyma.13g162100	27767517	27768046	-	_
			Glyma.13g162200	27768047	27772562	-	Translocase of chloroplast 90
			Glyma.13g162300	27773991	27778719	-	_
			Glyma.13g162400	27781636	27783846	-	Sarscorona virus main proteinase/severe acute respiratory syndrome coronavirus mainprotease
			Glyma.13g162600	27790437	27793621	-	Monogalactosyldiacyl glycerolsynthase/uridinediphosphogalactose-1,2-diacylglycerol galactosyl transferase
			Glyma.13g162700	27796825	27800728	-	_
			Glyma.13g162800	27817824	27822630	+	Inorganic diphosphatase/pyrophosphate phosphohydrolase
			Glyma.13g162900	27823310	27833374	+	Anion transporter6, chloroplastic-related
	AX-90405818<br>AX-90405799<br>AX-90496747		*Glyma.13g163000*	27833892	27837459	-	Heparanase-likeprotein3
			Glyma.13g163100	27847463	27849868	-	_
			Glyma.13g163200	27850927	27852651	+	Adenosine triphosphatase/ triphosphatase
			Glyma.13g163300	27854463	27856435	-	_
			Glyma.13g163400	27861309	27869162	+	Ankyrin repeat and dhhc-type zn-finger domain containing proteins
			Glyma.13g163500	27876748	27886905	+	Protein phosphatase1, regulatory subunit, and related proteins/ leucine rich repeat proteins, some proteins contain f-box
			Glyma.13g163600	27887744	27892434	-	Tpr repeat containing protein
			Glyma.13g163700	27895777	27898359	-	UPF0051 protein abci8, chloroplastic-related
			Glyma.13g163800	27896301	27896661	+	_
			Glyma.13g163900	27905208	27908859	-	EFH and domain family a1,a2-related
			Glyma.13g164000	27911603	27912972	+	_

			Glyma.13g164100	27913120	27916690	-	Major facilitator super family protein
			Glyma.13g164200	27922878	27924801	-	Bi functional arginine demethylase and lysyl-hydroxylasejmjd6
			Glyma.13g164300	27934139	27936052	-	_
			Glyma.13g164400	27940695	27943408	+	_
			Glyma.13g164500	27944852	27945076	+	_
			Glyma.13g164600	27952816	27953422	-	_
			Glyma.13g164700	27958127	27961278	-	_
	AX-90521967	Gm13	*Glyma.13g164800*	27967025	27969167	-	Platz transcription factor family protein
			Glyma.13g164900	27975423	27977287	+	Domain of unknown function (duf1817)
			Glyma.13g165000	27987286	27988809	+	_
			Glyma.13g165100	27991996	27994467	+	Glutamate receptor3.4-related
			Glyma.13g165200	27997373	27999343	+	40sribosomal proteins 11 family member
	AX-90336511	Gm13	*Glyma.13g165300*	28011615	28015224	+	Protein phosphatase2c
			Glyma.13g165400	28016508	28021594	-	116kdau 5 small nuclear ribonucleo protein component
			Glyma.13g165500	28030788	28034710	+	Ring finger domain-containing
			Glyma.13g165600	28035507	28038063	-	_
	AX-90502945	Gm16	*Glyma.16g173700*	33430875	33438490	+	Aaaatpase
			Glyma.16g173800	33431688	33431888	-	_
			Glyma.16g173900	33439127	33447439	-	Leucine richr epeat (lrr_1)/ (lrrnt_2)/ (lrr_8)
			Glyma.16g174000	33452980	33456674	-	lrr_1/llr_8
			Glyma.16g174100	33475023	33478604	-	lrrnt_2/ lrr_8
			Glyma.16g174200	33491255	33494665	+	Micro tubule-severing ATPase/ katanin
			Glyma.16g174300	33493635	33496282	-	Histone deacetylase/hdac
			Glyma.16g174400	33529239	33531183	+	lrr_1
			Glyma.16g174500	33532929	33535530	+	lrr_1/llr_8
			Glyma.16g174600	33541866	33550018	+	lrr_1/ llr_2/llr_8

			Glyma.16g174700	33568260	33573288	+	lrr_1/ llr_2/llr_8
			Glyma.16g174800	33588132	33591504	+	lrr_1/llr_8
			Glyma.16g174900	33595320	33596393	-	Aaaatpase
			Glyma.16g175000	33606851	33614825	+	lrr_1/ llr_2/llr_8
			Glyma.16g175100	33620772	33629405	+	lrr_1/ llr_2/llr_8
	AX-90422071	Gm18	*Glyma.18g107000*	12028945	12032564	-	Ribose-phosphate pyrophospho kinase4
			Glyma.18g107100	12047167	12050021	+	Ancient ubiquitous protein1
			Glyma.18g107200	12059922	12060860	-	Peptidase of plants and bacteria (bsp)
			Glyma.18g107300	12091200	12095291	-	Carbohydrate-binding x8 domain-containing protein
			Glyma.18g107400	12099175	12100330	-	_
			Glyma.18g107500	12116972	12118952	+	Mitochondrial transcription termination factor family protein-related
			Glyma.18g107600	12164801	12170095	-	Protein kinase family protein
			Glyma.18g107700	12207661	12208644	-	Gag-polypeptide of ltrcopia-type (ubn2_3)
			Glyma.18g107800	12208710	12212011	-	_
			Glyma.18g107900	12220182	12260477	+	Callose synthase7-related
			Glyma.18g108000	12221736	12225257	-	Dnahelicasepif1/rrm3
**LAL**	AX-90523253	Gm02	*Glyma.02g237600*	42597212	42600960	-	Metal tolerance protein c3-related
			Glyma.02g237400	42580305	42582359	+	Leucine-rich repeat-containing protein
			Glyma.02g237500	42582609	42591402	-	B3DNA binding domain (b3)
			Glyma.02g237700	42602537	42604779	-	Protein of unknown function (duf2741)
			Glyma.02g237800	42607230	42611324	-	RNA binding protein related
			Glyma.02g237900	42615776	42617153	-	Protein of unknown function (duf1191)
			Glyma.02g238000	42627584	42631716	+	_
			Glyma.02g238100	42635741	42643194	-	Nicotinate-nucleotide diphosphorylase (carboxylating)/quinolinate phosphoribosyl transferase (decarboxylating)

			Glyma.02g238200	42645706	42649807	-	UDPglucuronate:xylanalpha-glucuronosyl transferase3-related
			Glyma.02g238300	42672255	42689914	-	ATP-binding cassette sub-family Bmember6, mitochondrial
			Glyma.02g238400	42692884	42703545	-	Cohesin loading factor (cohesin_load)
			Glyma.02g238500	42709015	42712333	-	Uncharacterized conserved protein
			Glyma.02g238600	42714308	42718195	-	CT120 protein
			Glyma.02g238700	42722617	42726326	-	Inosine-5-monophosphate dehydrogenase related
			Glyma.02g238800	42735396	42738350	+	Oxalyl-coadecarboxylase/oxalyl-coacarboxy-lyase
			Glyma.02g238900	42742465	42743004	-	_
			Glyma.02g239000	42747173	42750040	+	_
			Glyma.02g239100	42754798	42755899	+	Late embryogenesis abundant (lea) hydroxyproline-rich glycol protein-related
			Glyma.02g239200	42757315	42763576	-	Nibrin-related
	AX-90367890<br>AX-90407903<br>AX-90372917	Gm02	*Glyma.02g239300*	42777266	42779243	-	Acyl-activating enzyme5, peroxisomal-related
			Glyma.02g239400	42784584	42786251	-	Acyl-activating enzyme5, peroxisomal-related
			Glyma.02g239500	42799982	42802509	-	Acyl-activating enzyme5, peroxisomal-related
			Glyma.02g239600	42813400	42824492	-	Auxin response factor8
			Glyma.02g239700	42839384	42841491	-	40sribosomal proteins26
			Glyma.02g239800	42848299	42849703	+	_
			Glyma.02g239900	42853091	42855600	+	CPG binding protein
	AX-90456562	Gm05	*Glyma.05g224900*	40366096	40370669	-	_
			Glyma.05g223000	40248224	40250093	+	1-aminocyclopropane-1-carboxylate synthase7
			Glyma.05g223100	40259388	40262641	+	Mitochondrial ATP synthaseg subunit (ATP-synt_g)

			Glyma.05g223200	40264775	40268360	+	Regulator of vps4 activity protein-related
			Glyma.05g223300	40270196	40271323	+	O-methyl transferase-related
			Glyma.05g223400	40273312	40274992	+	Caffeoyl-coao-methyl transferase/ trans-caffeoyl-coa3-o-methyl transferase
			Glyma.05g223500	40275557	40278080	-	40sribosomal proteins15a-2-related
			Glyma.05g223600	40277423	40279367	+	_
			Glyma.05g223700	40283806	40293124	+	Insulinase (peptidasefamilym16) protein
			Glyma.05g223800	40294131	40295090	+	60sribosomal protein l31
			Glyma.05g223900	40297233	40299773	+	_
			Glyma.05g224000	40300268	40303288	-	Asparagine-trnaligase, mitochondrial-related
			Glyma.05g224100	40304087	40305165	+	_
			Glyma.05g224200	40307329	40312025	+	SNF7-related
			Glyma.05g224300	40316985	40318520	-	Myb-like DNA binding protein
			Glyma.05g224400	40325845	40327778	-	Zinc fingerc-x8-c-x5-c-x3-htype (andsimilar) (zf-ccch)
			Glyma.05g224500	40342583	40345336	+	Inositoloxygenase/myo-inositoloxygenase
			Glyma.05g224600	40345642	40345824	+	_
			Glyma.05g224700	40351005	40361888	+	Glycine-rich protein
			Glyma.05g224800	40362481	40363717	-	Clathrin coatassembly protein
			Glyma.05g225000	40376407	40381768	+	Mitochondrial processing peptidase/processing enhancing peptidase
			Glyma.05g225100	40387813	40393404	+	Nac domain containing protein75-related
			Glyma.05g225200	40398178	40400609	-	Protein-serine/threonine phosphatase/serine/threonine specific protein phosphatase//protein-tyrosine-phosphatase/ptpase

			Glyma.05g225300	40408912	40410821	+	Extensin-likeprotein-related
			Glyma.05g225400	40412601	40415905	-	Protein of unknown function(duf1005)
			Glyma.05g225500	40422630	40426560	+	Der1-likeprotein, derlin
			Glyma.05g225600	40427955	40429788	+	Profilin
			Glyma.05g225700	40432149	40439631	+	Profilin
			Glyma.05g225800	40441668	40445293	-	Glucanendo-1,3-beta-glucosidase12-related
	AX-90365252	Gm07	*Glyma.07g077700*	7078817	7101299	+	Leucine-rich repeat-containing protein
			Glyma.07g077800	7101306	7102021	+	_
			Glyma.07g077900	7116774	7117750	+	_
			Glyma.07g078000	7121636	7126695	+	Leucine-rich repeat-containing protein
			Glyma.07g078100	7126750	7135963	+	Leucine rich repeat proteins, some proteins contain f-box
			Glyma.07g078200	7144509	7148052	-	Armadillo/beta-catenin repeat-containing protein-related
			Glyma.07g078300	7150800	7154478	-	Alternative splicing factor srp55/b52/srp75 (rrm superfamily)
			Glyma.07g078400	7152903	7153630	+	_
			Glyma.07g078500	7152959	7153210	-	Subtilisin-like serineproteas
			Glyma.07g078600	7158821	7160401	-	Shnshine, DNA binding/transcription
			Glyma.07g078700	7175548	7177380	+	Xyloglucan:xyloglucosyl transferase/xyloglucanendo transglycosylase
			Glyma.07g078800	7182128	7184201	+	Ring finger domain-containing
			Glyma.07g078900	7187362	7192149	+	Protein-ribulosamine3-kinase/ribulosamine/erythrul osamine3-kinase
			Glyma.07g079000	7193037	7202151	-	Response regulator of two-compon
			Glyma.07g079100	7199099	7205256	-	_
			Glyma.07g079200	7200488	7201018	+	_
			Glyma.07g079300	7201131	7201427	+	_
			Glyma.07g079400	7204711	7206782	+	_
			Glyma.07g079500	7214161	7217333	+	_

			Glyma.07g079600	7237783	7238601	+	Lysm domain (lysm)
			Glyma.07g079700	7248737	7256522	+	Arsenite-transporting ATPase/arsenite-translocating ATPase
			Glyma.07g079800	7259786	7263265	+	_
			Glyma.07g079900	7266622	7267156	-	Pthr 22811//pthr22811:sf68-trans membrane emp24 domain
	AX-90477018	Gm07	*Glyma.07g080400*	7312327	7319873	-	Phospholipased beta1-related
			Glyma.07g080500	7328383	7331416	-	Subtilisin-like serine protease-related
			Glyma.07g080600	7336252	7339584	+	Disease resistance family protein/ lrr family
			Glyma.07g080700	7341451	7345688	-	Protein kinase domain (pkinase)/s-locusglycop
			Glyma.07g080800	7358782	7367281	+	Acyltransferase(acyltransferase)/ef
			Glyma.07g080900	7381339	7405785	+	Mads-box protein soc1
			Glyma.07g081000	7423408	7424268	-	ABC transporter transmembrane region
			Glyma.07g081100	7424545	7425990	-	Cyclin-a2-1-related
			Glyma.07g081200	7427658	7428245	-	Maintenance of killer16 mak16 protein-related
			Glyma.07g081300	7442510	7449341	-	Agamous-like MADS-box protein agl13-related
			Glyma.07g081400	7465769	7471829	+	Protein y45f10a.7, isoforma
			Glyma.07g081500	7472362	7475246	+	Sap18
			Glyma.07g081600	7479732	7487620	+	Polygalacturonate4-alpha-galacturonosyltra
	AX-90491184	Gm11	*Glyma.11g093700*	7111708	7115757	+	Set1/ash2 histone methyl transferase complex sub unit
			Glyma.11g093800	7116433	7123292	-	ATP-binding cassette transporter
			Glyma.11g095000	7206492	7209711	-	Glucanendo-1,3-beta-d-glucosidase/laminarinase
			Glyma.11g093900	7130214	7137304	-	Sf247-ATP-binding cassette transporter
			Glyma.11g094000	7140746	7145587	-	_
			Glyma.11g094100	7144509	7145178	+	_

			Glyma.11g094200	7147965	7152190	+	Biotin synthase
			Glyma.11g094300	7153148	7168980	-	Isoleucine-tRNA ligase/iso leucyl-trna synthetase
			Glyma.11g094400	7170437	7174192	-	28s ribosomal proteins9, mitochondrial
			Glyma.11g094500	7176300	7183060	-	_
			Glyma.11g094600	7187192	7190048	-	_
			Glyma.11g094700	7190632	7195838	-	Hydroxymethyl bi lanesynthase /uroporphyrinogen synthetase
			Glyma.11g094800	7199274	7201137	+	_
			Glyma.11g094900	7202266	7203515	-	Glucanendo-1,3-beta-d-glucosidase/laminarinase
			Glyma.11g095100	7212438	7214276	-	Glucanendo-1,3-beta-d-glucosidase/laminarinase
			Glyma.11g095200	7218860	7221194	-	Glucanendo-1,3-beta-d-glucosidase/laminarinase
			Glyma.11g095300	7222140	7235534	+	DNA jhomolog sub family c member
			Glyma.11g095400	7236082	7240527	-	Saccharo pinedehydrogenase NADP binding domain
			Glyma.11g095500	7242440	7249074	-	Adaptinc-terminal domain (alpha_adaptinc2)
			Glyma.11g095600	7249880	7254364	-	Hexokinase-3-related
			Glyma.11g095700	7263413	7265368	-	Ubiquitin-conjugating enzyme E2
			Glyma.11g095800	7267458	7279596	-	Nucleosome-remodeling factor subunit BPTF
			Glyma.11g095900	7287276	7288134	+	Probable lipid transfer (ltp_2)
			Glyma.11g096000	7294150	7296623	-	Gras domain family(gras)
			Glyma.11g096100	7299741	7307551	-	Proteins-acyl transferase 19-related
	AX-90348822<br>AX-90337775<br>AX-90498877	Gm11	*Glyma.11g096200*	7315127	7325455	+	4-amino butyrate--2-oxoglutarate transaminase/gamma-amino-n-butyra
			Glyma.11g096300	7325956	7326700	-	_
	AX-90443985<br>AX-90340335	Gm11	*Glyma.11g209100*	30057154	30061089	+	Monodehydro ascorbate reductase, cytoplasm
			Glyma.11g209200	30063770	30065301	-	60s acidic ribosomal proteinp2

			Glyma.11g209300	30097729	30105137	+	6-dioxo-6-phenylhexa-3-enoatehydrolase/ hohpdahydrolase
			Glyma.11g209400	30105649	30106749	-	Germin-like protein sub family 3 member
			Glyma.11g209500	30131080	30134126	+	Protein terminal flower1
			Glyma.11g209600	30147295	30159083	+	Protease u48 caaxprenyl protease rce1
			Glyma.11g209700	30163931	30165878	-	Protein little zipper3
			Glyma.11g209800	30180779	30182096	+	Purple acid phosphatase 21-related
			Glyma.11g209900	30188618	30192948	-	Zinc finger five domain containin GP
			Glyma.11g210000	30206429	30207913	-	An1-type zinc finger protein
			Glyma.11g210100	30223531	30231651	+	Transcription initiation factort FIID subunit12
			Glyma.11g210200	30234588	30237609	+	Lysm domain (lysm)
			Glyma.11g210300	30238668	30248611	-	Tri hydroxy ptero carpandi methyl allyl transferase/glyceollin synthase
	AX-90362827<br>AX-90513611<br>AX-90396575	Gm18	*Glyma.18g104500*	11526580	11531654	-	Plant mobile domain (pmd)
			Glyma.18g104600	11541115	11551882	-	Serine/threonine-protein phosphatase 5
			Glyma.18g105000	11665406	11665918	+	_
			Glyma.18g104700	11598036	11603174	+	Helicase (herpes_helicase)/ pif1-like helicase
			Glyma.18g104800	11608502	11608894	-	_
			Glyma.18g104900	11656641	11665405	+	_
			Glyma.18g105100	11680805	11684730	+	Leucine-rich repeat- containing protein
			Glyma.18g105200	11694516	11696250	+	Leucine-rich repeat-containing protetein
			Glyma.18g105300	11696911	11697669	+	Leucine-richrepeat-containing protein
			Glyma.18g105400	11702349	11704712	-	Hva22-like prote
			Glyma.18g105500	11705137	11710003	-	Protein huellenlos
			Glyma.18g105600	11715984	11723202	+	Bed finger-related
			Glyma.18g105700	11731323	11732124	+	Endonuclease/ exonuclease/ phosphatase family
**TRL**	AX-90431861	Gm06	*Glyma.06g040900*	3084559	3086167	-	Heat stress transcription factorb-4

			Glyma.06g041000	3104225	3111827	-	Ring finger domain-containing
			Glyma.06g041100	3118193	3119768	-	_
			Glyma.06g041200	3121756	3122364	-	Transferase family (transferase)
			Glyma.06g041300	3122900	3124901	-	Ransferase family (transferase)
			Glyma.06g041400	3132564	3136289	-	Transferase family(transferase)
			Glyma.06g041500	3139829	3144620	+	Atp-dependent protease cereblon
			Glyma.06g041600	3145139	3145351	-	_
			Glyma.06g041700	3147768	3152655	-	RNA recognition motif
			Glyma.06g041800	3161791	3163327	-	Wuschel-related homeo box4
			Glyma.06g041900	3169816	3171166	-	_
			Glyma.06g042000	3178447	3178644	-	_
			Glyma.06g042100	3179113	3180503	-	Ethylene-responsive transcription factored f008
			Glyma.06g042200	3180855	3181761	+	_
			Glyma.06g042300	3194307	3197140	+	Ranbinding protein 9-related
			Glyma.06g042400	3212730	3225119	+	Kinesin-like protein-related
			Glyma.06g042500	3226378	3229165	-	Serine/threonine protein kinase/tgf-betastimulated factor
			Glyma.06g042600	3233956	3236966	+	Cysteine protease component of protease
			Glyma.06g042700	3240841	3243050	-	Serine/threonine-protein kinase wnk
			Glyma.06g042800	3246839	3248053	-	F-box protein skip2
			Glyma.06g042900	3264037	3266176	+	Cycline
	AX-90446460<br>AX-90414551<br>AX-90472866	Gm11	*Glyma.11g055600*	4200285	4203022	-	Remorin,c-terminal region (remorin_c)
			Glyma.11g055700	4207710	4216150	+	Zeaxanthine poxidase, chloroplastic
			Glyma.11g055800	4220955	4221185	-	_
			Glyma.11g055900	4221420	4223125	-	_
			Glyma.11g056000	4234567	4239763	-	Swap mRNA splicing regulator
			Glyma.11g056100	4246705	4248880	+	Protein of unknown function (duf1685)
			Glyma.11g056200	4254959	4257382	+	Heat stress transcription factor B-1
			Glyma.11g056300	4255281	4255406	+	_
			Glyma.11g056400	4259181	4260890	+	RNA helicase

			Glyma.11g056500	4265079	4266558	+	Ef hand(ef-hand_1)/ ef hand(ef-hand_5)
			Glyma.11g056600	4272400	4273766	+	Emb
			Glyma.11g056700	4282555	4284383	-	Cyclin-dependent kinase inhibitor 6
			Glyma.11g056800	4296965	4298290	+	Methionine amino peptidase1d, mitochondrial
			Glyma.11g056900	4298795	4300993	-	Ca2+-independent phosphor lipasea2
			Glyma.11g057000	4304933	4309655	+	Sulfo transferase domain (sulfotransfer_1)
			Glyma.11g057100	4313411	4314027	+	_
			Glyma.11g057200	4320409	4321206	+	_
			Glyma.11g057300	4326263	4326902	+	_
			Glyma.11g057400	4332503	4333352	+	_
			Glyma.11g057500	4341698	4347460	+	Uncharacterized conserved protein
			Glyma.11g057600	4352469	4357731	+	Protein of unknown function, duf547 /leucine-zip
			Glyma.11g057700	4358078	4359945	-	Transducin/wd40 domain-containing protein
			Glyma.11g057800	4361981	4364146	+	17-beta-estradiol17-dehydrogenase/estrogen17-oxidoreductase
			Glyma.11g057900	4367145	4369966	-	Sf2-sap domain-containing protein
			Glyma.11g058000	4370574	4376076	-	Serine/threonine-protein kinase
			Glyma.11g058100	4384247	4386065	-	Peroxidase/lactoperoxidase
			Glyma.11g058200	4390109	4390700	-	_
			Glyma.11g058300	4392607	4397689	-	Mitogen-activated kinase kinasekinas
			Glyma.11g058400	4409851	4413688	+	Protein of unknown function duf260
			Glyma.11g058500	4417029	4423791	-	Tubulin-relatedprotein
			Glyma.11g058600	4425323	4427642	-	Myb family transcription factor
**NF**	AX-90428520<br>AX-90467878<br>AX-90468823<br>AX-90412442	Gm08	*Glyma.08g081500*	6152552	6161571	-	Histidin oldehydrogenase hdh
			Glyma.08g080000	6076298	6077881	+	Berberine bridge enzyme-like protein-related
			Glyma.08g080100	6079968	6081557	-	Berberine bridge enzyme-related

			Glyma.08g080200	6084010	6085893	+	Tetrahydro berberine oxidase/ thboxidase
			Glyma.08g080300	6086733	6088550	+	Ppr repeat (ppr)/ dyw family of nucleic acid deaminases
			Glyma.08g080400	6092714	6096079	+	Sf34-f22m8.11protein-related
			Glyma.08g080500	6097769	6099931	+	Sf34-f22m8.11protein-related
			Glyma.08g080600	6115848	6117996	+	Berberine bridge enzyme-related
			Glyma.08g080700	6119523	6122290	+	Berberine bridge enzyme-related
			Glyma.08g080800	6123319	6124310	-	_
			Glyma.08g080900	6127342	6129511	+	Berberine bridge enzyme-related
			Glyma.08g081000	6133267	6136646	-	Fatty acid hydroxylase
			Glyma.08g081100	6137956	6140938	-	Tubulinbeta-4chain-related
			Glyma.08g081200	6142155	6143076	+	Cupin domain (cupin_2)
			Glyma.08g081300	6145003	6149493	-	N-acetyl glucosaminyl phosphatidyl inositoldeacetylase/ n-acetylglucosa
			Glyma.08g081400	6152131	6155753	+	Protein phosphatase 2c28-related
			Glyma.08g081600	6162265	6170670	-	Hen1
			Glyma.08g081700	6174443	6177152	-	Stressup-regulated nod19 (surnod19)
			Glyma.08g081800	6178646	6180153	-	Stressup-regulated nod19 (surnod19)
			Glyma.08g081900	6182127	6185682	-	Protein pmt-1,isoformb
			Glyma.08g082000	6188267	6192585	-	26s proteasomenon-ATPase regulatory subunit8
			Glyma.08g082100	6196059	6199325	+	Programmed cell death protein5
			Glyma.08g082200	6202794	6210874	+	Potassium inward rectifier (kir)-like channe l3
			Glyma.08g082300	6206872	6207344	+	_
			Glyma.08g082400	6229164	6231818	+	Wrky transcription factor 28-related
			Glyma.08g082500	6244916	6252372	+	O-acetyl transferase cas1p-like protein-related
			Glyma.08g082600	6256587	6266032	+	Putative dynamitin
			Glyma.08g082700	6266414	6268400	-	U6s nrna-associated protein
		Gm08	*Glyma.08g093300*	7072042	7076041	-	Tartrate-resistant acid phosphatase type5

			Glyma.08g093400	7084753	7087202	+	Hetero dimeric geranyl geranyl pyro phosphates
			Glyma.08g093500	7088454	7092027	+	Tartrate-resistant acid phosphatase type5
			Glyma.08g093600	7092593	7098413	+	Tartrate-resistant acid phosphatase type5
			Glyma.08g093700	7097206	7101441	-	Procollagen-proline3-dioxygenase/ prolyl3-hydroxylase
			Glyma.08g093800	7102857	7109102	-	Hydroxyproline-rich glycoprotein-like protein
			Glyma.08g093900	7120394	7123346	-	Xylo glucanendo transglucosylase
			Glyma.08g094000	7139990	7141988	+	Transmembrane ascorbate ferri reductase3-rel
			Glyma.08g094100	7145070	7149534	-	Proteinoberon3
			Glyma.08g094200	7157662	7163180	-	Early growth response protein-related
	AX-90493535<br>AX-90455156<br>AX-90331992		*Glyma.08g094300*	7160799	7160957	+	_
			Glyma.08g094400	7174551	7178950	-	Protein kinase domain (pkinase)/wall-associated
			Glyma.08g094500	7184460	7192472	+	Protein of unknown function (duf789)
			Glyma.08g094600	7195107	7198806	-	Enolase superfamily, mandel ateracemase
			Glyma.08g094700	7205721	7208341	-	Mitochondrial 28s ribosomal proteins28
			Glyma.08g094800	7209255	7213386	-	Non-specific serine/ threonine protein kinase
			Glyma.08g094900	7218371	7222854	-	IFA-binding protein
			Glyma.08g095000	7230311	7239978	-	Clathrin assembly protein
			Glyma.08g095100	7242895	7244309	+	NADH dehydrogenase trans membrane subunit
			Glyma.08g095200	7245023	7250047	-	Uncharacterized conserved protein
			Glyma.08g095300	7258432	7260587	-	Proteinagamous-like82
			Glyma.08g095400	7267235	7270875	+	Iso amylacetate-hydrolyzin gesterase and related enzymes

	AX-90365804	Gm09	*Glyma.09g051100*	4454515	4461881	+	Cellulose synthase acatalytic subunit7
			Glyma.09g050200	4367844	4370056	+	Cationic amino acid transporter5
			Glyma.09g050300	4373865	4377763	+	Protein kinase domain (pkinase)
			Glyma.09g050400	4379081	4379851	-	_
			Glyma.09g050500	4380780	4383807	-	PPR repeat (ppr)/ppr repeat family (ppr_2)
			Glyma.09g050600	4384885	4387181	-	Cationic amino acid transporter 5
			Glyma.09g050700	4401152	4405156	-	Halo acid dehalogenase-like hydrolas
			Glyma.09g050800	4413615	4418223	-	All antoate deiminase
			Glyma.09g050900	4447546	4450090	+	Poly galacturonase QRT3
			Glyma.09g051000	4452259	4453424	+	_
			Glyma.09g051200	4464563	4473730	-	Choline-phosphatecytidylyltransferase/phosphorylcholinetransferase
			Glyma.09g051300	4470781	4470999	+	_
			Glyma.09g051400	4480786	4483499	-	S-adenosyl-l-methionine-dependentmethyltrans
			Glyma.09g051500	4488096	4493102	-	Protein trigalactosyl di acylglycerol 4
			Glyma.09g051600	4494692	4498211	-	Calicylin binding protein
			Glyma.09g051700	4494923	4495081	+	_
			Glyma.09g051800	4499012	4503649	-	Eukaryotic mitochondrial regulator protein (bot1p)
			Glyma.09g051900	4508892	4509626	-	Vqmotif(vq)
			Glyma.09g052000	4519979	4527492	-	E1-e2atpase(e1-e2_atpase)/ heavy-metal-
			Glyma.09g052100	4536612	4538089	+	60s ribosomal protein l27
			Glyma.09g052200	4540284	4557984	+	Set domain (set)/gyf domain (gyf)
			Glyma.09g052300	4557593	4557820	+	_
	AX-90495283<br>AX-90449059<br>AX-90520390<br>AX-90434197<br>AX-90498061	Gm09	*Glyma.09g073400*	7732397	7733253	-	Auxin responsive protein (auxin_inducible)
			Glyma.09g072600	7590260	7593045	+	40s ribosomal proteins 3a
			Glyma.09g072700	7619132	7620402	-	Pectin esterase/pectin methyl esterase
			Glyma.09g072800	7633793	7634182	-	_

	AX-90419697<br>AX-90492156<br>AX-90493039		*Glyma.09g072900*	7645379	7646352	+	Myb/sant-likedna-binding domain (myb_dna-bind_3)
			Glyma.09g073000	7650927	7651406	-	_
			Glyma.09g073100	7654381	7657822	-	Predicted transposase
			Glyma.09g073200	7697529	7708882	-	Respiratory burst oxidase homolog protein
			Glyma.09g073300	7723202	7726373	+	Auxin-responsive protein-like protein-related
			Glyma.09g073500	7756930	7758261	+	Vacuolar sorting protein35
			Glyma.09g073600	7809852	7816248	-	Sucrose synthase1-related
**NT**	AX-90522644<br>AX-90466067<br>AX-90467825	Gm01	*Glyma.01g219800*	54913356	54934465	-	Lysine-specificdemethylaselid
			Glyma.01g218100	54819025	54824457	+	Myosin heavy chain-related protein
			Glyma.01g218200	54828960	54830170	-	Lurp-one-related (lor)
			Glyma.01g218300	54838494	54842120	+	Deoxy nucleotidyl transferase terminal
			Glyma.01g218400	54842601	54846844	+	Kdellys-asp-glu-leucontaining-rela
			Glyma.01g218500	54847794	54848478	-	Expressedprotein
			Glyma.01g218600	54849376	54854956	-	Expressedprotein
			Glyma.01g218700	54857259	54858579	-	_
			Glyma.01g218800	54860809	54866642	+	Protein tyrosine kinase (pkinase_tyr)/ leucine rich repeat
			Glyma.01g218900	54866798	54868389	-	Actin-depolymerizingfactor10-related
			Glyma.01g219000	54870113	54875022	+	E1-e2atpase(e1-e2_atpase)/ heavy-metal-associated
			Glyma.01g219100	54880250	54888021	+	E1-e2atpase(e1-e2_atpase)/heavy-metal-
			Glyma.01g219200	54889915	54895017	+	C2calcium/lipid-binding plant phosphoribosylt
			Glyma.01g219300	54895767	54897277	+	Exo stosin family protein-related
			Glyma.01g219400	54898589	54899940	+	Glutathione peroxidase
			Glyma.01g219500	54901381	54901854	+	Glutathione peroxidase8-related
			Glyma.01g219600	54903061	54907533	-	At hook motif DNA-binding family protein

			Glyma.01g219700	54908697	54911774	-	Poly nucleotidyl transferase/ ribonuclease H-lik
			Glyma.01g219900	54935370	54941060	+	Glutamate—cysteine ligase
			Glyma.01g220000	54942535	54944623	+	RNAse prpr2/ rpp21/ snm1 subunit domain(rpr2)
			Glyma.01g220100	54947015	54948375	+	Bromo-adjacent homology (bah) domain-containing
			Glyma.01g220200	54950379	54954980	+	Bromo-adjacent homology (bah) domain-containing
			Glyma.01g220300	54959627	54963856	+	duf3527
			Glyma.01g220400	54964945	54971202	+	Signalpeptidepeptidase-like4
			Glyma.01g220500	54969086	54969580	+	_
			Glyma.01g220600	54974523	54976556	+	Aquaporinpip1-4-related
			Glyma.01g220700	54977695	54985676	-	Mitogen-activated kinase kinase
			Glyma.01g220800	54988418	54991271	-	_
			Glyma.01g220900	55000110	55006202	+	Tir domain (tir_2)
			Glyma.01g221000	55007371	55009590	-	Ring finger containing protein
			Glyma.01g221100	55018468	55020621	-	Sf20-cctmotiffamilyprotein
			Glyma.01g221200	55022862	55025351	+	Plac8family(plac8)
			Glyma.01g221300	55027025	55028244	-	Anaphase-promoting complex(apc), subunit11
	AX-90430136<br>AX-90419830<br>AX-90317331	Gm09	*Glyma.09g073700*	7863209	7879959	-	Inositol5-phosphatase
			Glyma.09g073800	7897323	7901496	-	Zinc finger five domain containin gpr
	AX-90313864<br>AX-90431604<br>AX-90382601<br>AX-90312113<br>AX-90455909<br>AX-90306078	Gm09	*Glyma.09g073900*	7920912	7922111	-	Expressed protein
			Glyma.09g074000	7924556	7927259	-	Zinc finger five domain containin gpr
			Glyma.09g074100	7943646	7949050	+	Red protein IK factor cytokine
	AX-90325580<br>AX-90370286<br>AX-90457815	Gm13	*Glyma.13g263800*	36698350	36700879	-	Oxidoreductase, 2og-feiioxygenasef
			Glyma.13g261800	36568871	36570396	+	11-oxo-beta-amyrin30-oxidase/cyp72a154

	AX-90452276<br>AX-90480597<br>AX-90305162<br>AX-90433497<br>AX-90383650		*Glyma.13g261700*	36553556	36556696	-	11-oxo-beta-amyrin30-oxidase/cyp72a154
			Glyma.13g261900	36571819	36576038	+	Protease family S26 mitochondrialinn
			Glyma.13g262000	36579260	36585256	-	11-oxo-beta-amyrin30-oxidase/cyp72a154
			Glyma.13g262100	36586108	36589827	-	11-oxo-beta-amyrin30-oxidase/cyp72a154
			Glyma.13g262200	36591312	36595829	-	Hepta prenyl diphosphate synthase
			Glyma.13g262300	36592394	36593638	+	_
			Glyma.13g262400	36600186	36601991	-	Carbohydrate-binding x8 domain-containing protein
			Glyma.13g262500	36603843	36608523	+	Chalcone isomerase
			Glyma.13g262600	36612366	36614107	-	Purinepermease11-related
			Glyma.13g262700	36617625	36621135	+	Emb
			Glyma.13g262800	36625422	36628081	+	Emb
			Glyma.13g262900	36635322	36637117	+	Emb
			Glyma.13g263000	36643870	36645875	+	Fbd(fbd)
			Glyma.13g263100	36651160	36653640	+	Fbd (fbd)
			Glyma.13g263200	36652023	36663332	-	O-methyltransferase
			Glyma.13g263300	36664541	36668259	+	Ribokinase
			Glyma.13g263400	36671175	36675044	+	Ribokinase
			Glyma.13g263500	36676213	36676962	+	_
			Glyma.13g263700	36687392	36688292	-	_
			Glyma.13g263600	36687392	36692071	+	Mitogen-activated protein kinase18-related
			Glyma.13g263900	36705555	36706942	-	Oxidoreductase, 2og-feii oxygenase
			Glyma.13g264000	36707745	36709746	-	Codeine3-o-demethylase/code in eo-demethylase
			Glyma.13g264100	36711934	36719667	-	Nucleo porin auto peptidase
			Glyma.13g264200	36723747	36728882	-	Aceto acetatede carboxylase
			Glyma.13g264300	36734670	36739644	-	Sodium/bile acid co transporter 4
			Glyma.13g264400	36740782	36744087	-	Rag1-activating protein1

			Glyma.13g264500	36755174	36759150	+	Rhamnose biosynthetic enzyme1-related
			Glyma.13g264600	36759779	36772020	-	_

Table S4. List of Genes showed higher expression in root organ/tissue.

Gene Name	Start	End	Strand	Description
Glyma.02g250200	43779470	43781548	+	Protein phosphatase 2c3-related
Glyma.02g250400	43786923	43791698	-	Methyl transferase pmt10-related
Glyma.06g126100	10315437	10317265	-	Abscisicacid receptor pyl10-related
Glyma.06g127500	10462640	10469529	+	Splicing factor 3a subunit3
Glyma.06g127700	10486250	10492229	+	Plastid-lipid-associated protein 14,chloroplastic-related
Glyma.14g199400	46435759	46438392	-	Disease resistance protein rpp13-related
Glyma.18g300500	57841316	57843280	+	Pirin
Glyma.18g301700	57945537	57947130	-	Brassinosteroid insensitive1-associated receptor kinase1-related
Glyma.18g302100	57988925	57992117	+	Cdp-diacylglycerol--glycerol-3-phosphate3-phosphatidyltransferase/phosphatidylglycerophosphatesynthase
Glyma.03g251500	44723994	44729654	-	Serine/threonine-protein phosphatase pp1isozyme2-related
Glyma.03g251600	44735415	44736486	-	Peptidyl-prolylcis-transisomerase cyp18-3-related
Glyma.03g253100	44880486	44882438	+	Beta catenin-related armadillo repeat-containing
Glyma.06g127800	10493847	10506001	-	Histone-lysine n-methyl transferase atx4-related
Glyma.12g193500	35498865	35503832	-	Alcohol dehydrogenase related
Glyma.13g162200	27768047	27772562	-	Translocase of chloroplast90, chloroplastic
Glyma.13g163000	27833892	27837459	-	Heparanase-like protein3
Glyma.13g163500	27876748	27886905	+	Protein phosphatase1, regulatory subunit, and related proteins//leucine rich repeat proteins, some proteins contain f-box
Glyma.13g163700	27895777	27898359	-	UPF0051protein abci8, chloroplastic-related
Glyma.02g237800	42607230	42611324	-	RNA-binding protein-related
Glyma.02g239100	42754798	42755899	+	Late embryogenesis abundant(lea) hydroxyproline-richglycoprotein-related
Glyma.05g223500	40275557	40278080	-	40s ribosomal proteins 15a-2-related
Glyma.05g224500	40342583	40345336	+	Inositol oxygenase/myo-inositol oxygenase
Glyma.05g225300	40408912	40410821	+	Extensin-like protein-related
Glyma.05g225700	40432149	40439631	+	Profilin
Glyma.07g078200	7144509	7148052	-	Armadillo/beta-catenin repeat-containing protein-related
Glyma.07g079900	7266622	7267156	-	Pthr22811/pthr22811:sf68-transmembrane emp24domain-containi
Glyma.07g080500	7328383	7331416	-	Subtilisin-like serine protease-related
Glyma.07g080600	7336252	7339584	+	Disease resistance family protein/lrr family protein

Glyma.07g081600	7479732	7487620	+	Polygalacturonate4-alpha-galacturonosyl related
Glyma.11g095600	7249880	7254364	-	Hexokinase-3-related
Glyma.11g095700	7263413	7265368	-	Ubiquitin-conjugating enzyme E2
Glyma.11g095800	7267458	7279596	-	Nucleosome-remodeling factor subunit BPTF
Glyma.11g209100	30057154	30061089	+	Mono dehydro ascorbate reductase, cytoplasm
Glyma.11g209200	30063770	30065301	-	60s acidic ribosomal protein P2
Glyma.11g209400	30105649	30106749	-	Germin-like protein subfamily 3 member 2
Glyma.11g209600	30147295	30159083	+	Protease u48caaxprenyl protease RCE1
Glyma.08g082000	6188267	6192585	-	26s proteasome non-atpase regulatory subunit 8
Glyma.08g082100	6196059	6199325	+	Programmedcelldeathprotein5
Glyma.08g082200	6202794	6210874	+	Potassium inward rectifier (kir)-like channel 3
Glyma.08g082500	6244916	6252372	+	O-acetyl transferase CAS1p-like protein-related
Glyma.08g093400	7084753	7087202	+	Hetero dimeric geranyl pyrophosphates
Glyma.08g093900	7120394	7123346	-	Xylo glucan endo trans glucosylase/hydrolase
Glyma.08g095400	7267235	7270875	+	Isoamyl acetate-hydrolyzing esterase and related enzymes
Glyma.09g051100	4454515	4461881	+	Cellulose synthase a catalytic subunit7 [udp-fo
Glyma.09g051900	4508892	4509626	-	Vqmotif (vq)
Glyma.09g052000	4519979	4527492	-	E1-e2atpase (e1-e2_atpase)
Glyma.09g073300	7723202	7726373	+	Auxin-responsive protein-like protein-related
Glyma.01g218100	54819025	54824457	+	Myosin heavy chain-related protein
Glyma.01g218300	54838494	54842120	+	De oxynucleotidyl transferase terminal-intera
Glyma.01g218400	54842601	54846844	+	Kdellys-asp-glu-leu containing-related
Glyma.01g220600	54974523	54976556	+	Aqua porin PIP1-4-related
Glyma.13g261700	36553556	36556696	-	11-oxo-beta-amyrin30-oxidase/cyp72a154
Glyma.13g261900	36571819	36576038	+	Protease family S26 mitochondrial
Glyma.13g262000	36579260	36585256	-	11-oxo-beta-amyrin30-oxidase/cyp72a154
Glyma.13g263600	36687392	36692071	+	Mitogen-activated protein kinase18-related

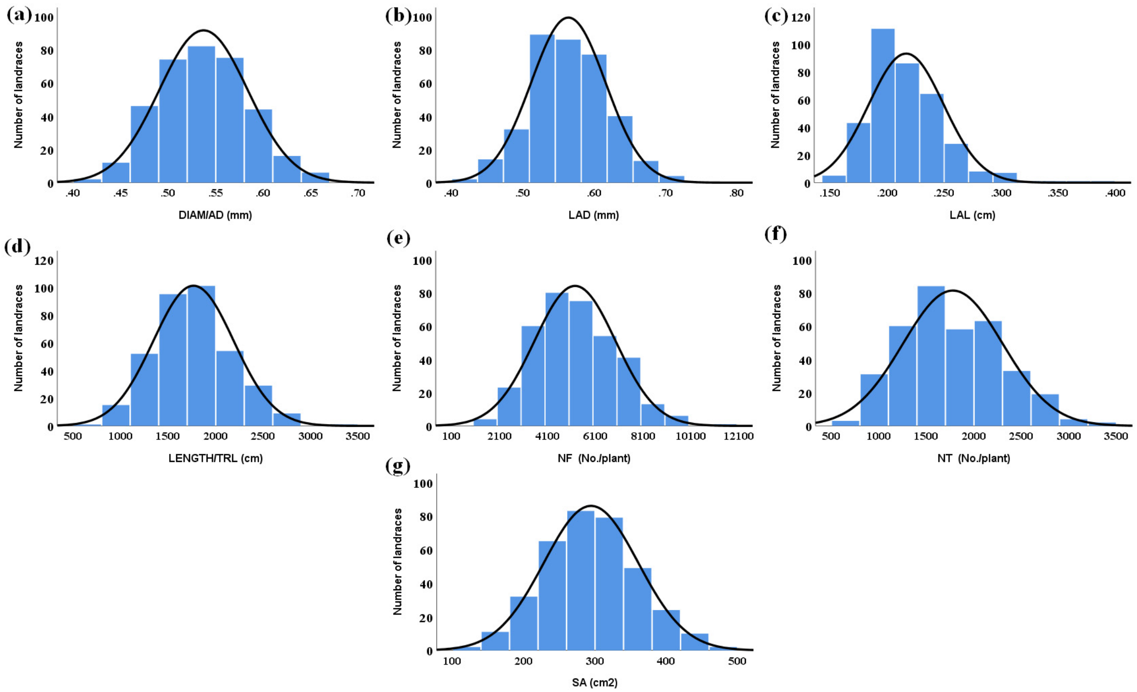

Figure S1. Frequency distribution of root traits among 357 soybean landraces. (a) Average diameter (DIAM), (b) Link average diameter (LAD), (c) Link average length (LAL), (d) Total root length (LENGTH), (e) Number of forks (NF), (f) Number of tips (NT), and (g) Surface area (SA).

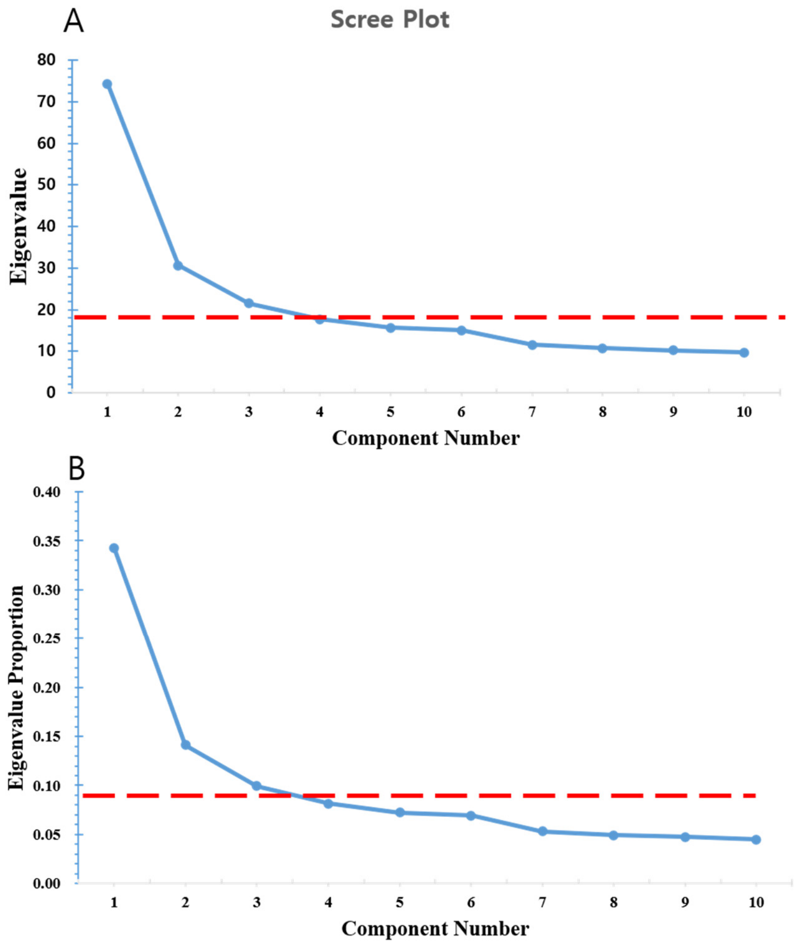

Figure S2. Representation of scree plot of principal component analysis. (A) The number of principal components (PCs) is on the x-axis and the associated eigenvalues are on the y-axis. (B)The number of principal components (PCs) is on the x-axis and the associated eigenvalue proportion is on the y-axis. The optimal number of principal components to explain the variation found in the genotype is found by visually determining the largest point of inflection or "elbow" of the non-linear red trend line.

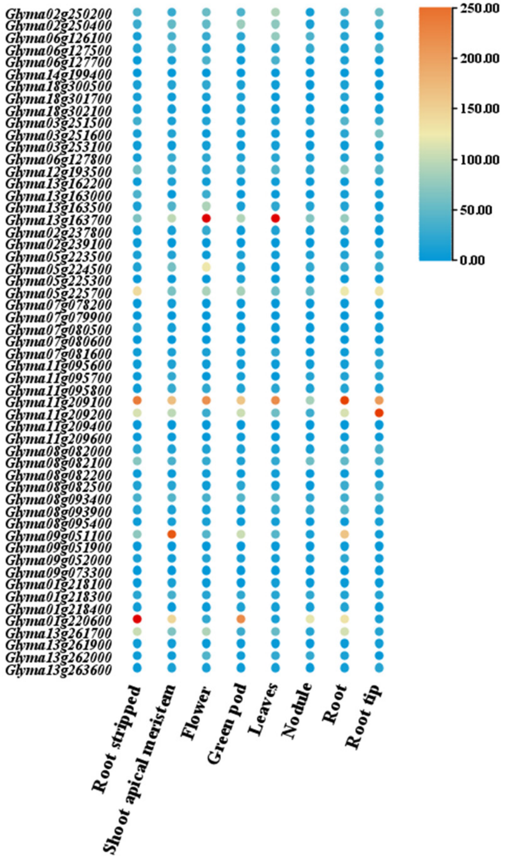

Supplemental Figure S3. Heatmap representing the expression of 55 candidate genes in various soybean plant tissue types. Data were accessed from [ePlant (https://bar.utoronto.ca/eplant_soybean/)](https://bar.utoronto.ca/eplant_soybean/) database. The level of expression showed differential patterns for selected genes.

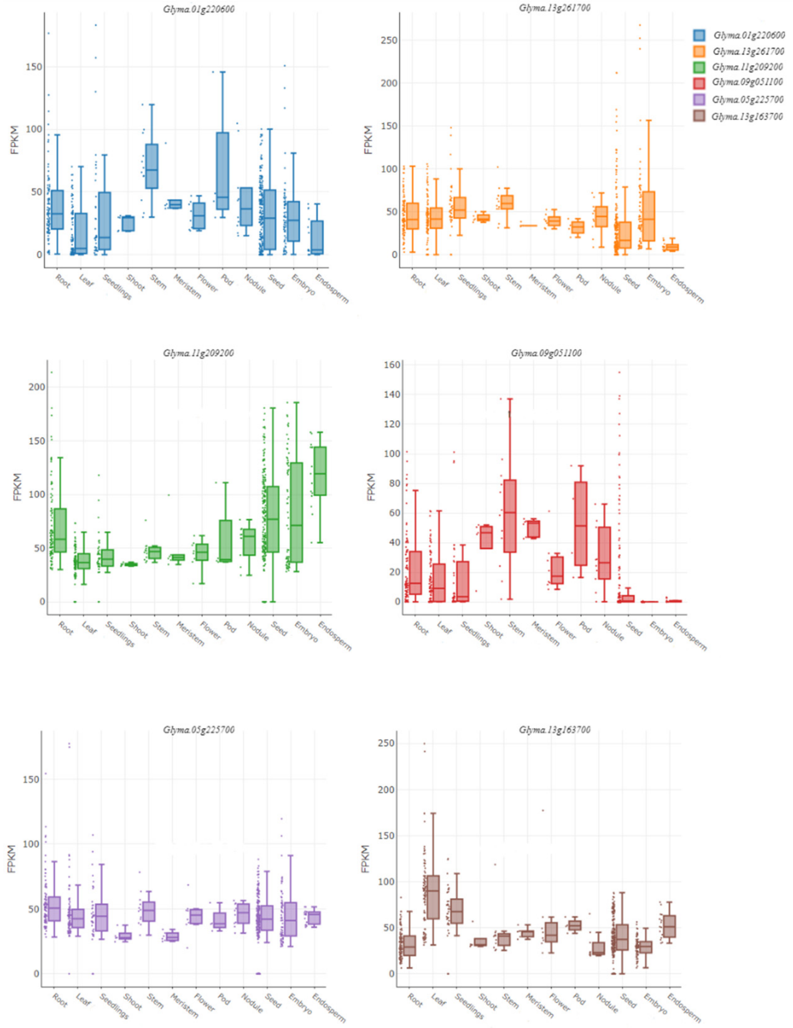

Figure S4. Candidate genes expression levels across 12 different tissues.

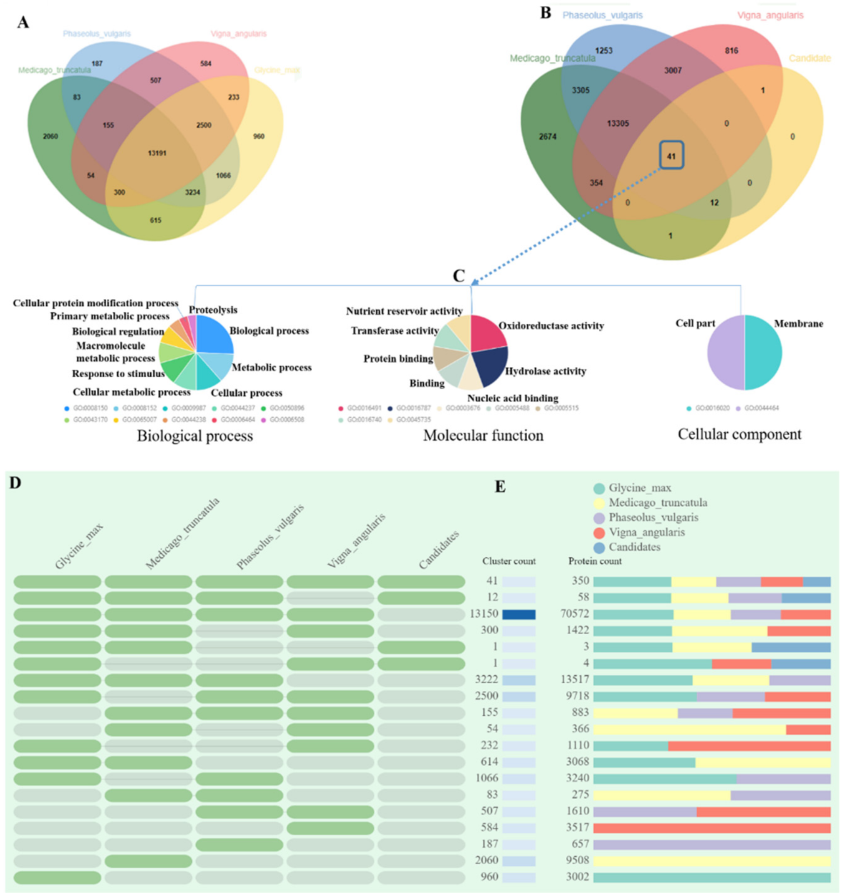

Figure S5. Comparative genome analysis of 55 candidate genes expressed in root tissue (A). Venn diagram representing the core orthologs and specific genes cluster for *Glycine max*, *Medicago truncatula, Phaseolus vulgaris* and *Vigna augularis*. (B). Venn diagram representing the core orthologs and specific genes cluster for candidate genes of candidate genes, *Medicago truncatula, Phaseolus vulgaris and Vigna augularis*. (C). Gene ontology term enrichment analysis of core candidate genes (D). Shared gene clusters of orthologous groups categories. (E). Protein families count shared between *Glycine max, Medicago truncatula, Phaseolus vulgaris and Vigna augularis*, and candidate genes.
